# Evaluation of a Mobile-Based Immunization Decision Support System for Scheduling Age-Appropriate Vaccine Schedules for Children Younger Than 2 Years in Pakistan and Bangladesh: Lessons From a Multisite, Mixed Methods Study

**DOI:** 10.2196/40269

**Published:** 2023-02-17

**Authors:** Danya Arif Siddiqi, Rozina Feroz Ali, Mubarak Taighoon Shah, Vijay Kumar Dharma, Anokhi Ali Khan, Tapash Roy, Subhash Chandir

**Affiliations:** 1 IRD Global Singapore Singapore; 2 IRD Pakistan Karachi Pakistan; 3 IRD Bangladesh Dhaka Bangladesh

**Keywords:** missed opportunities for vaccination, mobile-based immunization decision support system, catch-up immunizations

## Abstract

**Background:**

Missed opportunities for vaccination (MOVs), that is, when children interact with the health system but fail to receive age-eligible vaccines, pose a crucial challenge for equitable and universal immunization coverage. Inaccurate interpretations of complex catch-up schedules by health workers contribute to MOVs.

**Objective:**

We assessed the feasibility of a mobile-based immunization decision support system (iDSS) to automatically construct age-appropriate vaccination schedules for children and to prevent MOVs.

**Methods:**

A sequential exploratory mixed methods study was conducted at 6 immunization centers in Pakistan and Bangladesh. An android-based iDSS that is packaged in the form of an application programming interface constructed age-appropriate immunization schedules for eligible children. The diagnostic accuracy of the iDSS was measured by comparing the schedules constructed by the iDSS with the gold standard of evaluation (World Health Organization–recommended Expanded Programme on Immunization schedule constructed by a vaccines expert). Preliminary estimates were collected on the number of MOVs among visiting children (caused by inaccurate vaccination scheduling by vaccinators) that could be reduced through iDSS by comparing the manual schedules constructed by vaccinators with the gold standard. Finally, the vaccinators’ understanding, perceived usability, and acceptability of the iDSS were determined through interviews with key informants.

**Results:**

From July 5, 2019, to April 11, 2020, a total of 6241 immunization visits were recorded from 4613 eligible children. Data were collected for 17,961 immunization doses for all antigens. The iDSS correctly scheduled 99.8% (17,932/17,961) of all age-appropriate immunization doses compared with the gold standard. In comparison, vaccinators correctly scheduled 96.8% (17,378/17,961) of all immunization doses. A total of 3.2% (583/17,961) of all due doses (across antigens) were missed in age-eligible children by the vaccinators across both countries. Vaccinators reported positively on the usefulness of iDSS, as well as the understanding and benefits of the technology.

**Conclusions:**

This study demonstrated the feasibility of a mobile-based iDSS to accurately construct age-appropriate vaccination schedules for children aged 0 to 23 months across multicountry and low- and middle-income country settings, and underscores its potential to increase immunization coverage and timeliness by eliminating MOVs.

## Introduction

### Background

Routine childhood immunization is the cornerstone of an efficient public health system and childhood disease prevention. Despite progress in improving routine immunization coverage, in 2022, 17% and 7% of children aged 0 to 23 months in Pakistan and Bangladesh, respectively, did not receive the third dose of the diphtheria-tetanus-pertussis vaccine [1]. The problem is further exacerbated for children who do visit health facilities in low- and middle-income countries (LMICs), but the immunization system fails to provide all age-appropriate vaccines to one in every 3 of these children [2]. This creates missed opportunities for vaccination (MOVs), where despite being eligible for vaccination, a child is not administered one or more of the vaccine doses [3]. MOVs remain a rampant problem in many LMICs [4,5], leading to underimmunization and delayed vaccination of children.

A systematic review of MOVs across LMICs showed a pooled MOV prevalence of 32.2% (95% CI 26.8%-37.7%) among children and 46.9% (95% CI 29.7%-64.0%) among women of childbearing age [2]. MOV prevalence estimates are even higher for some countries, ranging from 42% to 89% [6,7]. A study from Pakistan reported that despite being eligible for the pentavalent vaccine (Penta) at the measles vaccine visit, 34% of children did not receive the required dose of Penta even though they visited the immunization clinic and were vaccinated for measles [8]. Lack of awareness among parents and providers is one of the primary causes of MOVs, with vaccinator confusion about contraindications and the immunization schedule being the main factors [2,9]. In the absence of frequent refresher training, improper health care worker practices lead to repeated MOVs in LMICs and high-income countries [10,11]. However, regardless of the amount of training, scheduling age-appropriate vaccinations, especially catch-up schedules (when the child has missed or delayed one or more vaccines), is a complex task [12,13]. A study from Illinois showed that 33% of all health workers constructed incorrect hypothetical catch-up schedules in a survey designed to determine health workers’ knowledge of catch-up immunizations [10]. In LMICs, where vaccinators are more likely to be overburdened and pressed for time [14], the likelihood of making errors in scheduling is even higher.

Decision support systems have proven to be powerful tools for improving clinical care and patient outcomes across various domains [15,16]. They have contributed to improving evidence-based medical practices, reducing medical errors, and improving adherence to standard care practices [17,18]. At the basic level, an immunization decision support system (iDSS) provides patient-specific vaccination recommendations at each immunization visit, considering the child’s date of birth and vaccination history and ensuring the appropriate scheduling of feasible doses.

Currently, examples of iDSS deployment are limited and almost exclusively constitute complex systems from high-income country settings. For example, in the United States, clinical decision support systems based on national standard guidelines for immunization are embedded in regional immunization information systems [19] or web-based immunization forecasting services accessible by various electronic health records [20]. There is a dearth of literature on contextually appropriate, efficient, and practical mobile-based iDSS that is easily implementable within LMICs.

### Objective

We aimed to analyze the diagnostic accuracy of an innovative mobile-based iDSS in an LMIC setting to construct age-appropriate vaccination schedules for children aged 0 to 23 months and compared with the gold standard of evaluation (World Health Organization [WHO]–recommended Expanded Programme on Immunization [EPI] schedule constructed by a vaccines expert). We also aimed to generate preliminary evidence for MOVs among visiting children (caused by inaccurate vaccination scheduling by vaccinators) that could be reduced through iDSS by comparing the manual schedules constructed by vaccinators with the gold standard. Finally, we reported the findings on the vaccinators’ understanding, perceived usability, and acceptability of the iDSS.

## Methods

### Study Design

We implemented a sequential exploratory mixed methods study design with a quantitative component preceding the qualitative interviews. In the quantitative component, the children’s demographic details and immunization history were recorded by trained study staff using iDSS. The iDSS used this information to formulate an age-appropriate immunization schedule for the children, and it was recorded on the back end and was not visible to the study staff (or vaccinators). Simultaneously, the study staff also captured the manually constructed immunization schedules determined by the vaccinators as indicated on the child’s government-issued immunization cards. Through this process, we were able to capture both the iDSS and vaccinator schedules simultaneously for the same child (antigen doses). We used this information to assess the diagnostic accuracy of the iDSS algorithm by comparing the age-appropriate immunization schedules constructed by the iDSS for children aged 0 to 23 months with the gold standard of evaluation (WHO-recommended EPI schedule constructed by a vaccine expert). We also independently compared the vaccine schedules constructed manually by the vaccinators for the same children with the gold standard. This allowed us to generate preliminary evidence of MOVs resulting from inaccurate vaccination schedules constructed by vaccinators. This phase was followed by a qualitative phase in which the vaccinators were provided with iDSS-enabled study phones. After vaccinators had a chance to use the iDSS, we conducted in-depth interviews with vaccinators at the participating immunization centers regarding their experience of using the iDSS, its perceived utility, and acceptability.

### Study Sites

We conducted a multicountry study at 6 immunization centers, 3 each in Pakistan and Bangladesh. Both countries vary in terms of full immunization coverage rate (Pakistan 66%; Bangladesh 84%) and infant mortality rates (81/1000 live births in Pakistan; 43/1000 live births in Bangladesh) [21,22]. Immunization centers were selected based on the influx of children, availability of vaccines and vaccinators, and absence of any kind of decision support system at these sites. The selected study sites had high penetration of mobile phones (>90%) and the presence of cellular networks (data connectivity) among the population [23,24].

Two of the selected immunization centers in Pakistan were located in Gilgit district in Gilgit Baltistan territory, which had a full immunization coverage rate of 57% among children aged 12 to 23 months in 2018 [21]. The third selected immunization center in Pakistan was a private center located in the Rahim Yar Khan district in Punjab province, with a full immunization coverage rate of 65% in 2019 [23].

In Bangladesh, all 3 immunization centers were located in Dhaka city in Dhaka district. The district had a full immunization coverage rate of 85% among children aged 12 to 23 months as of 2019 [25].

### Study Population

The inclusion criteria for the study were as follows: children must be aged <2 years, visiting any of the 6 selected immunization centers for routine vaccination, and presenting with an immunization card. Exclusion criteria were as follows: children visiting for immunization campaigns and those who did not receive vaccination during their visit. We obtained verbal consent from the caregivers of the eligible children before enrollment.

For the qualitative component, our study population included all vaccinators from the participating immunization centers who used iDSS app during the study. We obtained written consent from the study participants before the interviews.

### Ethics Approval

Ethics approval for the study was obtained from the Institutional Review Boards of the Interactive Research and Development (Pakistan; IRD_IRB_2019_01_010) and Building Resources Across Communities James P. Grant School of Public Health (Bangladesh).

### Vaccination Schedule

Pakistan’s routine EPI schedule in 2019 included BCG (bacille Calmette-Guérin) vaccine and oral polio vaccine (OPV) at birth; 3 doses of Penta (containing diphtheria-tetanus-pertussis, hepatitis B, *Haemophilus influenzae* type b vaccine), pneumococcal vaccine (PCV10), and OPV at 6, 10, and 14 weeks; 2 doses of rotavirus vaccine (Rota) at 6 and 10 weeks; a single dose of inactivated polio vaccine (IPV) at 14 weeks; and 2 doses of measles vaccine at 9 and 15 months.

For Bangladesh, the EPI schedule included BCG vaccine at birth; Penta, PCV, and OPV at 6, 10, and 14 weeks; IPV at 6 and 14 weeks; and measles-rubella (MR) vaccine at 9 and 15 months.

### Development of the iDSS

We developed an android-based iDSS designed for mobile-based deployment, packaged in the form of an application programming interface (API) to function both as a stand-alone module and interoperable with other digital applications or platforms, such as web-based or mobile-based electronic immunization registries (EIRs).

The iDSS was formulated as a 2-step process comprising a data entry form and display interface showing the proposed vaccination schedule. In the first step, the details of the child, including the date of birth and immunization history, are entered in the data entry form of the iDSS. The algorithm uses this information to construct an age-appropriate immunization schedule (including the vaccines due at the current visit and those to be scheduled) tailored to the child and the respective country’s EPI schedule.

**Figure 1:**
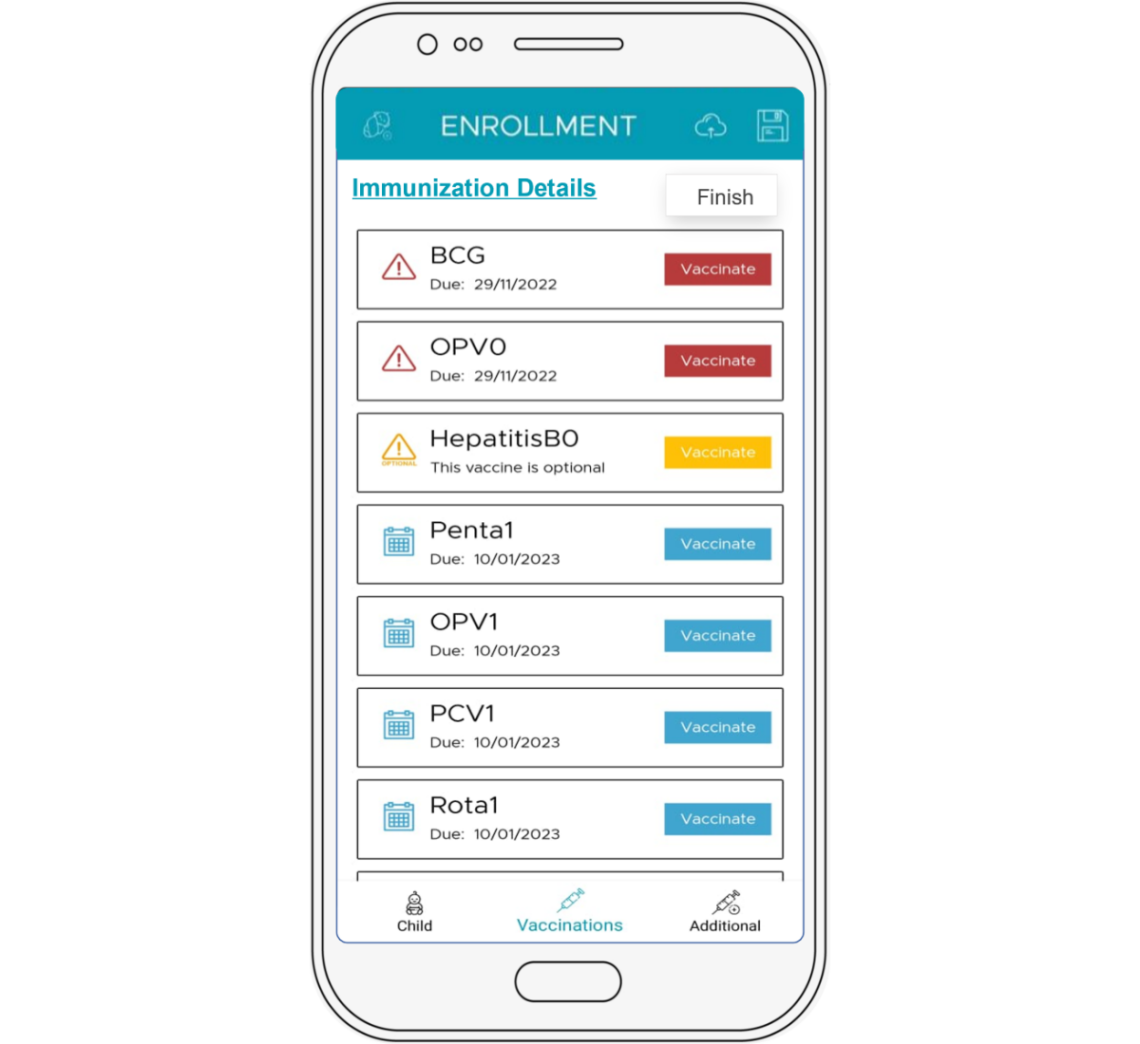
A screenshot showcasing features of the immunization decision support system app. BCG: bacille Calmette-Guérin; HepB: Hepatitis B; OPV: oral polio vaccine; PCV; pneumococcal vaccine; Penta: pentavalent vaccine; Rota: rotavirus vaccine.

iDSS automatically reformulates the child’s immunization schedule after every visit, adjusting for missed appointments and delayed vaccinations and considering the birth date, previous vaccines, standard interval based on live or inactivated vaccines and interdosing schedule, without the need for any manual calculation by the vaccinators. Changes in the EPI schedule and new vaccines are also incorporated into the iDSS algorithm. The results (current and scheduled vaccines) are instantly displayed on the iDSS interface through a color-coded system that allows for straightforward interpretation, especially by low literacy and overburdened vaccinators (Figure 1). The iDSS proposes the dates for scheduled vaccines (accounting for public holidays and vaccine-specific days at centers) and prompts the vaccinator through warning messages if vaccines are being administered out of schedule to ensure that interdose gap guidelines are followed.

In addition, the iDSS algorithm can be customized to the needs of the country, according to the respective EPI schedule. The iDSS is also multilingual and is developed as open-source software for easy integration and interoperability across a variety of settings and systems. As part of the iDSS development, the module was pretested in-house for quality assurance and with randomly selected vaccinators (outside the study site) for limited field deployment. A detailed overview of the iDSS is provided in Multimedia Appendix 1.

### Study Procedures and Data Collection

We enrolled eligible children whose accompanying caregivers provided verbal consent for participation. At enrollment, a unique study identifier was allocated to each child, and each follow-up visit by the child to the participating immunization center was recorded as a unique visit. Trained study staff used the iDSS installed on study mobile phones to record the demographics and immunization history of the enrolled children to enable iDSS to schedule their current and future vaccination visits. This information was recorded at the back end of the iDSS, and both the study staff and vaccinators were blinded to it. Simultaneously, the study staff also recorded the vaccination schedules determined by the vaccinators as per routine, which were obtained from the child’s immunization cards. Vaccinators did not use the iDSS during the quantitative phase of the study. The iDSS app was linked to a web-based dashboard that allowed the real-time downloading of data for further analysis.

For the qualitative component of the study, we interviewed 16 vaccinators (11 from Pakistan and 5 from Bangladesh) after obtaining written consent. These vaccinators were provided with mobile phones with iDSS that they could use to schedule current and future vaccinations. Before using the iDSS, vaccinators were given a 2-day training by the study staff on using the iDSS module as part of their daily immunization activities. At the end of the period when the vaccinators had used the iDSS for at least 4 weeks, the study team interviewed the vaccinators. In-person interviews were conducted by Project Managers at both sites, who were qualified public health researchers with a medical background. The interviews were facilitated by Research Associates, who helped to take notes. We used a 16-item semistructured interview guide covering user experience, acceptability, and feasibility of the iDSS feature, and suggestions for improvement. Demographic indicators, including age, education, gender, and years of experience, were also collected from the participants. Each interview lasted between 25 and 40 minutes and was audio-recorded and transcribed for further analysis.

### Study Outcomes

Our primary study outcome was the diagnostic accuracy of the iDSS in constructing age-appropriate vaccination schedules as per the WHO-recommended EPI guidelines. In addition, we examined the vaccinators’ perceived usability and acceptability of the iDSS. A secondary outcome included preliminary estimates of the number of MOVs among children aged 0 to 23 months visiting study immunization centers for routine immunization visits.

### Data Security

The phones used for data collection by the field staff and vaccinators had password locks with an additional level of protection through software sign-in passwords. Data collected through iDSS were uploaded to a secure database server. All the data sent from the phones to the server and back were encrypted in transit using the PBKDF2 algorithm (an industry standard). Access to data on the server was through a password-protected web dashboard interface, and only those involved with the project had access to the data for research purposes.

### Statistical Analysis

#### Quantitative Analysis

For descriptive analysis, we used frequencies and percentages for categorical data and means and SDs for continuous data. We compared the age-appropriate immunization doses scheduled by the iDSS and vaccinator with the gold standard. The latter helped determine the evidence for MOVs caused by inaccurate vaccination schedules constructed by vaccinators. To determine the gold standard vaccination schedule, an expert epidemiologist and practicing pediatrician reviewed the immunization history and date of birth or age of the child to determine the age-appropriate immunization doses due at each visit as per the WHO-recommended EPI schedule for the respective country. Summary statistics for the diagnostic tests were calculated using the *diagt* command package. Forest plots for sensitivity and specificity were generated using the *metan* command. The accuracy of the iDSS was determined along with the area under the receiver operating characteristic curves and their range. Analyses were performed using STATA software (version 17.0; StataCorp LLC).

#### Qualitative Analysis

The recordings of the qualitative data collected through the in-depth interviews were first transcribed and then translated into English. Transcriptions were coded by 2 researchers separately, who were public health practitioners, trained and experienced in performing qualitative analysis. The researchers extensively discussed and scrutinized the results to ensure the trustworthiness and comprehensiveness of the analysis. The final coding was shared with a third researcher to resolve any inconsistencies in the codes. Using a thematic analysis approach, the codes were sorted into categories to converge toward key overarching themes. Data were analyzed using NVivo qualitative data analysis software (version 12, 2018; QSR International).

## Results

### Quantitative Analysis

**Figure 2:**
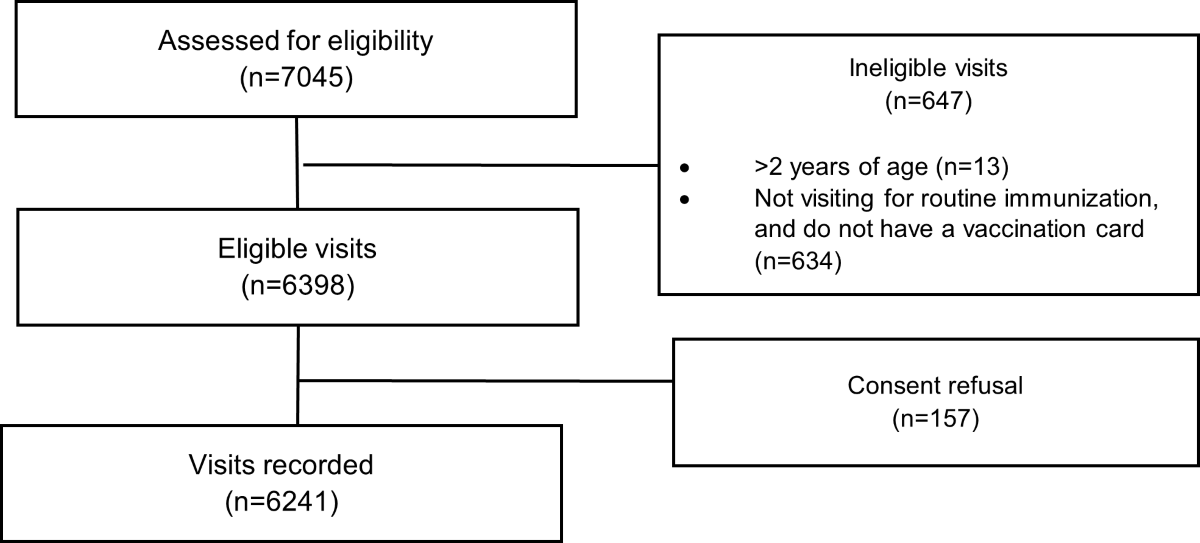
Study participant flow for all enrolled children and subsequent immunization visits from July 5, 2019, to April 11, 2020, in Pakistan and Bangladesh.

From July 5, 2019, to April 11, 2020, a total of 6241 immunization visits were recorded from 4613 eligible children. A total of 73% (4557/6241) of visits were recorded from 3 immunization centers in Pakistan, and 27% (1684/6241) of visits were recorded from 3 immunization centers in Bangladesh (Figure 2).

The proportion of male children (2435/4613, 52.8%) enrolled in the study was slightly higher than that of female children (2178/4613, 47.2%) across both the sites (Pakistan: 1720/3197, 53.8%) and Bangladesh (715/1416, 50.5%; Table 1). Most (3348/4613, 72.6%) children enrolled in the study were aged ≤6 months at the time of enrollment, both in Pakistan (2423/3197, 75.8%) and Bangladesh (925/1416, 65.3%).

Table 2 shows the age-appropriate immunization doses scheduled by both the iDSS and vaccinators compared with the gold standard. In Pakistan, we collected data on 13,039 immunization doses for all antigens. The iDSS correctly scheduled 99.8% (13,015/13,039) of all age-appropriate immunization doses that should have been administered at the current visit compared with the gold standard, ranging from 99.2% to 100% for BCG vaccine, OPV-0 to 3, Penta-1 to 3, PCV-1 to 3, Rota-1 to 2, measles vaccine 1, and IPV vaccine doses. Of all the antigens, the proportion of correctly scheduled doses by iDSS was the lowest at 96.7% (202/209) for the measles-2 vaccine. In comparison, the vaccinator correctly scheduled 96.2% (12,545/13,039) of all the immunization doses that should have been administered on the current visit compared with the gold standard, ranging from 97% to 100% for BCG vaccine, OPV-0 to 3, Penta-1 to 3, PCV-1 to 3, Rota-1 to 2, and measles-2. However, the proportion of correctly scheduled doses by vaccinators due on the current visit decreased to 94.5% (362/383) and 66.3% (653/985) for measles-1 and IPV vaccine doses, respectively. Owing to errors in the vaccination schedules constructed by vaccinators, 3.8% (494/13,039) of all due immunization doses were missed by the vaccinators. Among the age-eligible children, the highest proportion of MOVs was for the polio and measles vaccines, where 33.7% (332/985) and 5.5% (21/383) of all due immunization doses, respectively, were missed by vaccinators.

In Bangladesh, we recorded data on 4922 immunization doses. The iDSS correctly scheduled 99.9% (4917/4922) of the age-appropriate immunization doses that should have been administered on the current visit compared with the gold standard, with the lowest proportion of correctly scheduled doses for the measles-2 vaccine (196/201, 97.5%). In comparison, the vaccinator correctly scheduled 98.2% (4833/4922) of all immunization doses that should have been administered on the current visit, ranging from 97.5% to 100% for BCG vaccine, OPV-1 to 3, Penta-1 to 3, PCV-1 to 3, IPV-1, and measles-rubella-2, but dropped to 95.1% (137/144) for measles-rubella-1 and 87.4% (355/406) for IPV-2 vaccine doses. Owing to errors in the vaccination schedules constructed by vaccinators, 1.8% (89/4922) of all due immunization doses were missed by the vaccinators. Similar to the findings in Pakistan, the highest proportion of MOVs was for polio and measles-1 vaccines, where 12.6% (51/406) and 4.9% (7/144) of all due immunization doses, respectively, were missed by vaccinators.

**Figure 3:**
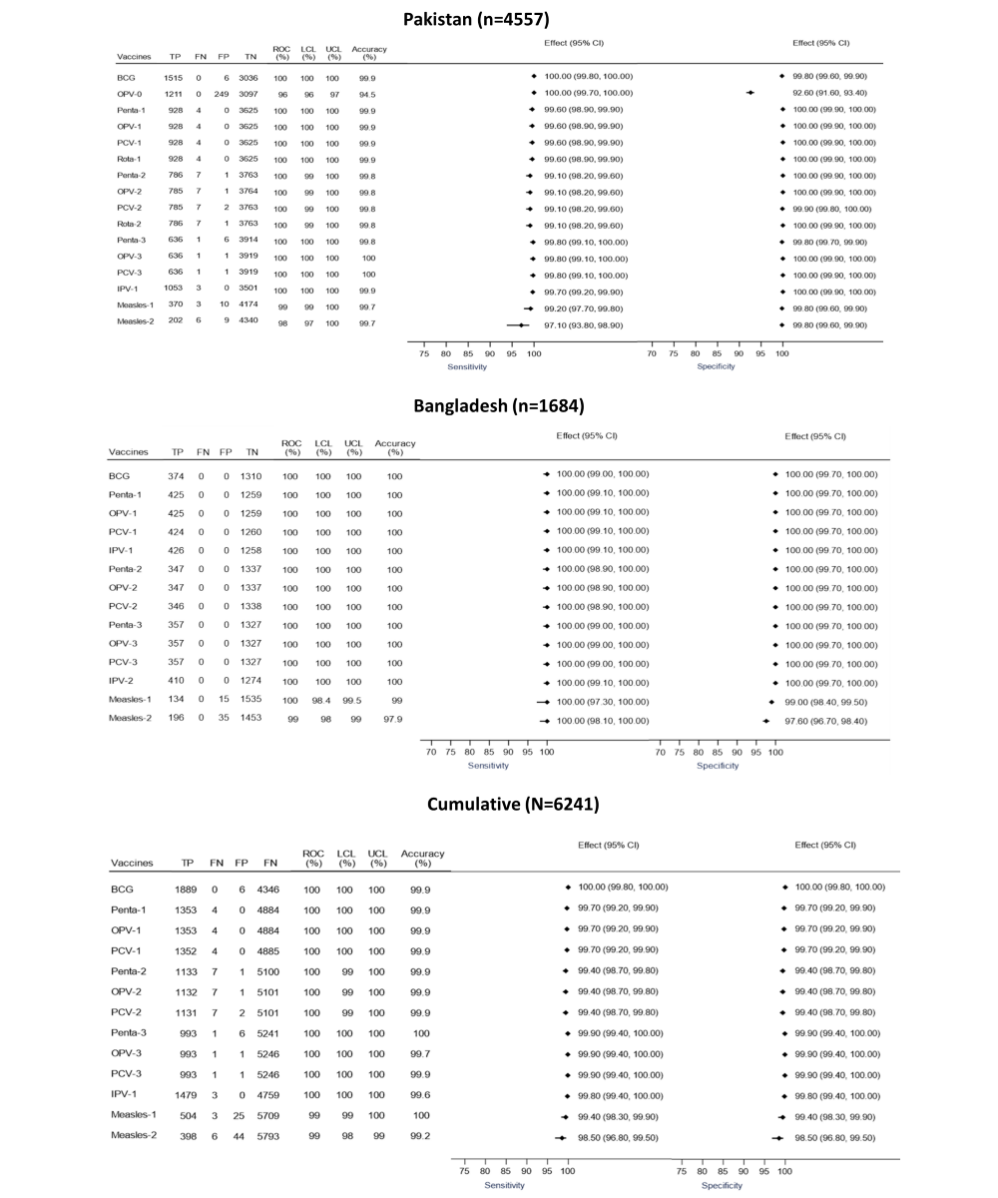
Diagnostic accuracy of vaccine schedules constructed by the immunization decision support system compared with the gold standard presented as a forest plot. BCG: bacille Calmette-Guérin; CI: Confidence Interval; FN: false negative; FP: false positive; LCL: lower confidence level; OPV: oral polio vaccine; PCV: pneumococcal vaccine; Penta: pentavalent vaccine; ROC: receiver operating curve; Rota: rotavirus vaccine; TP: true positive; TN: true negative; UCL: upper confidence level.

**Figure 4:**
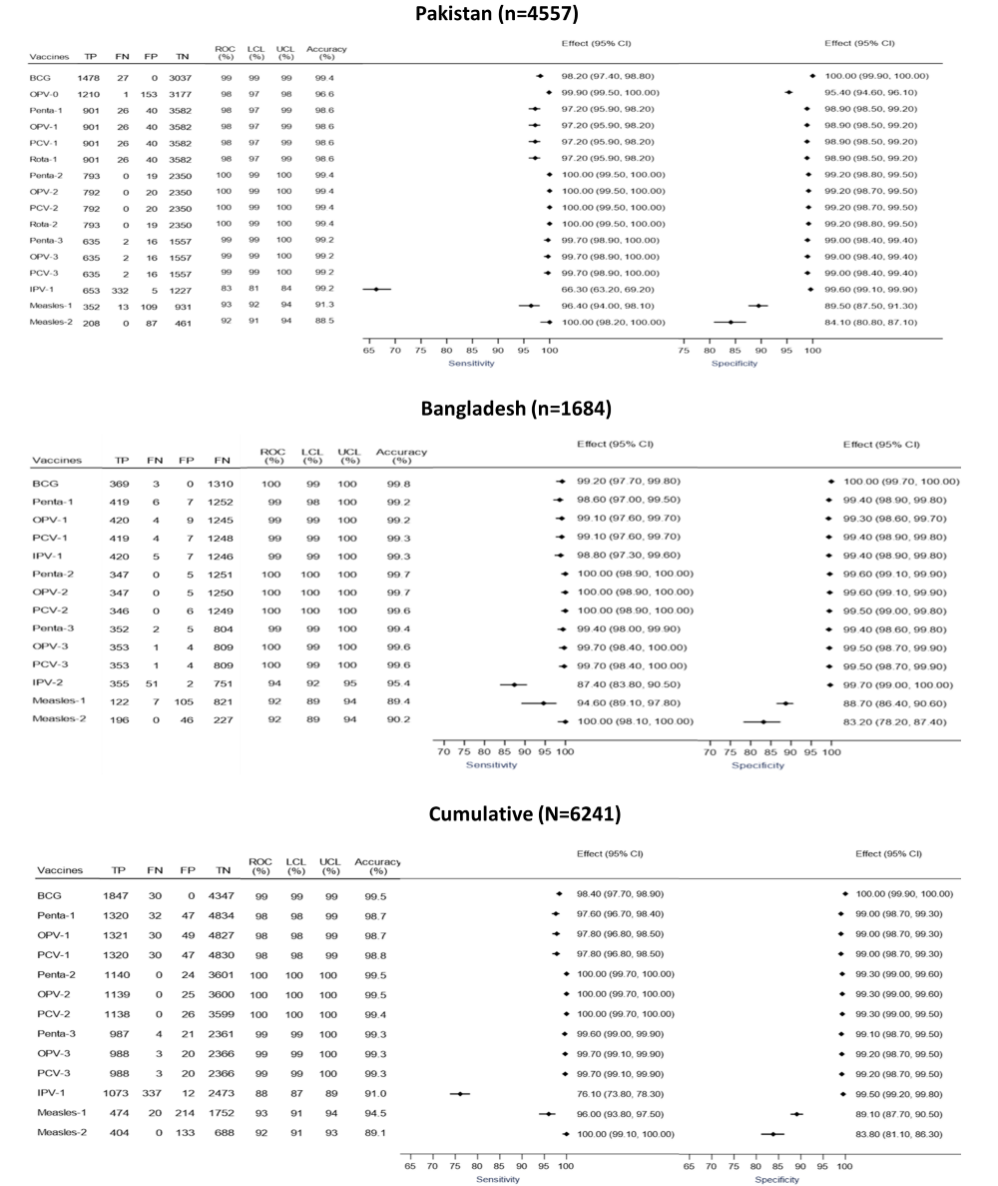
Diagnostic accuracy of manual vaccine schedules constructed by vaccinators compared with the gold standard presented as a forest plot. BCG: bacille Calmette-Guérin; CI: Confidence Interval; FN: false negative; FP: false positive; LCL: lower confidence level; OPV: oral polio vaccine; PCV: pneumococcal vaccine; Penta: pentavalent vaccine; ROC: receiver operating curve; Rota: rotavirus vaccine; TP: true positive; TN: true negative; UCL: upper confidence level.

Compared with the gold standard, in Pakistan, the accuracy of the iDSS varied between 94.5% and 100% across vaccines versus 88.5% to 99.4% for the immunization doses scheduled by the vaccinator. The iDSS demonstrated a sensitivity of 97.1% to 100%, whereas the sensitivity of the vaccinator’s scheduling was between 66.3% and 100%. The estimated specificity for iDSS compared with the gold standard was 92.6% to 100%, whereas the specificity for vaccinator scheduling was 84.1% to 100%. The results were similar for the Bangladesh site, with iDSS demonstrating a higher accuracy (97.9%-100%) and a higher sensitivity of 100% compared with the gold standard, whereas estimates of accuracy and sensitivity for vaccinator scheduling were 89.4% to 99.8% and 87.4% to 100%, respectively. The iDSS had a specificity of 97.6% to 100% compared with the gold standard, whereas the specificity for the vaccinator’s scheduling was 83.2% to 100% (Figures 3 and 4).

Across both the sites, receiver operating curve analysis showed that the iDSS had high overall accuracy of scheduling age-appropriate immunization doses (area under the curve ranging from 99%-100% across antigens), whereas for vaccinator schedules, estimates for area under the curve ranged between 88% and 100%.

On the basis of the findings of the diagnostic accuracy of the iDSS in which the sensitivity and specificity were <100% for selected antigens, we updated the iDSS algorithm by fixing the errors related to the vaccine intervals and recommended age for administering the vaccines based on the WHO guidelines.

**Table 1:** Characteristics of study participants enrolled in Pakistan and Bangladesh sites^a^.

Characteristics			Participants in Pakistan (n=3197)			Participants in Bangladesh (n=1416)			Total (N=4613)
Female, n (%)			1477 (46.2)			701 (49.5)			2178 (47.2)
**Enrollment age (months), n (%)**									
	≤6	2423 (75.8)			925 (65.3)			3348 (72.6)	
	6 to ≤12	462 (14.5)			235 (16.6)			697 (15.1)	
	12 to ≤18	284 (8.9)			242 (17.1)			526 (11.4)	
	18 to ≤24	28 (0.9)			14 (1)			42 (0.9)	
Father’s age (years), mean (SD)			32.7 (6.6)			33.3 (5.6)			32.9 (6.3)
**Father’s education (years), n (%)**									
	0	571 (17.9)			19 (1.3)			590 (12.8)	
	1-5	180 (5.6)			169 (11.9)			349 (7.6)	
	6-10	1018 (31.8)			289 (20.4)			1307 (28.3)	
	11-12	486 (15.2)			249 (17.6)			735 (15.9)	
	>13	942 (29.5)			690 (48.7)			1632 (35.4)	
**Father’s occupation, n (%)**									
	Government employee	1048 (32.8)			162 (11.4)			1210 (26.2)	
	Self-employed	821 (25.7)			518 (36.6)			1339 (29)	
	Daily wage earner	678 (21.2)			40 (2.8)			718 (15.6)	
	Private employee	337 (10.5)			689 (48.7)			1026 (22.2)	
	Others	313 (9.8)			7 (0.5)			320 (6.9)	
Mother’s age (years), mean (SD)			26.3 (5.0)			26.4 (4.8)			26.3 (4.9)
**Mother’s education (years), n (%)**									
	0	820 (25.6)			5 (0.4)			825 (17.9)	
	1-5	177 (5.5)			176 (12.4)			353 (7.7)	
	6-10	1024 (32)			373 (26.3)			1397 (30.3)	
	11-12	473 (14.8)			395 (27.9)			868 (18.8)	
	>13	703 (22)			467 (33)			1170 (25.4)	
**Mother’s occupation, n (%)**									
	Home maker	2806 (87.8)			1151 (81.3)			3957 (85.8)	
	Private employee	118 (3.7)			128 (9)			246 (5.3)	
	Government employee	115 (3.6)			97 (6.9)			212 (4.6)	
	Other	158 (4.9)			40 (2.8)			198 (4.3)	
**Ethnicity, n (%)**									
	Gilgiti	2700 (84.5)			N/A^b^			2.700 (58.5)	
	Saraiki	173 (5.4)			N/A			173 (3.8)	
	Balochi	94 (2.9)			N/A			94 (2)	
	Bangal	N/A			1413 (99.8)			1413 (30.6)	
	Other	230 (7.2)			3 (0.2)			233 (5.1)	
Household income (US $ per month), mean (SD)			267.3 (620.3)			442.8 (453.9)			321.1 (580)
**Enrollment vaccine, n (%)**									
	BCG^c^	1125 (35.2)		367 (25.9)			1492 (32.3)		
	OPV-0^d,e^	4 (0.1)		N/A			4 (0.1)		
	Penta-1^f,g^	484 (15.1)		107 (7.6)			591 (12.8)		
	Penta-2^h^	480 (15)		228 (16.1)			708 (15.3)		
	Penta-3^i^	381 (11.9)		247 (17.4)			628 (13.6)		
	IPV^j^	1 (0)		N/A			1 (0)		
	Measles-1	428 (13.4)		226 (16)			654 (14.2)		
	Measles-2	294 (9.2)		241 (17)			535 (11.6)		
**Age at enrollment vaccine (weeks), mean (SD)**									
	BCG	2.1 (3.2)		6.7 (2.9)			3.2 (3.7)		
	OPV-0	0.2 (0.2)		N/A			0.2 (0.2)		
	Penta-1^g^	9.0 (4.4)		8.2 (7.6)			8.8 (5.2)		
	Penta-2^h^	15.2 (5.8)		12.6 (4.1)			14.4 (5.5)		
	Penta-3^i^	21.3 (7.0)		18.8 (8.5)			20.4 (7.7)		
	IPV	35.1 (0.0)		N/A			35.1 (0.0)		
	Measles-1	42.0 (7.4)		41.2 (5.6)			41.7 (6.8)		
	Measles-2	68.1 (6.6)		67.6 (5.2)			67.9 (6.0)		

^a^Number of children enrolled in the study. A total of 4557 immunization visits were recorded from the Pakistan site and 1684 visits were recorded from the Bangladesh site.

^b^N/A: not applicable.

^c^BCG: bacille Calmette-Guérin.

^d^OPV: oral polio vaccine.

^e^Schedule for polio vaccine is different at both sites. Pakistan: OPV0-3 doses at birth, 6, 10, and 14 weeks and single IPV dose at 14 weeks; Bangladesh: OPV1-3 doses at 6, 10, and 14 weeks and 2 IPV doses at 6 and 14 weeks.

^f^Penta: pentavalent vaccine.

^g^Administered with PCV-1 and OPV-1 and Rota-1 and IPV-1 vaccines.

^h^Administered with PCV-2 and OPV-2 and Rota-2 vaccine.

^i^Administered with PCV-3 and OPV-3 and IPV-2 vaccine.

^j^IPV: inactivated polio vaccine.

**Table 2:** Antigen-wise doses scheduled by the gold standard (World Health Organization [WHO]–recommended Expanded Programme on Immunization [EPI] schedule)^a^, immunization decision support system (iDSS), and vaccinator for Pakistan and Bangladesh sites.

Vaccines	Pakistan (n=13,036)						Bangladesh (n=4920)						Total (n=17,956)^b^				
	Due as per gold standard, n	Due as per iDSS, n (%)		Vaccinated by vaccinator, n (%)		Due as per gold standard, n		Due as per iDSS, n (%)		Vaccinated by vaccinator, n (%)		Due as per gold standard, n		Due as per iDSS, n (%)		Vaccinated by vaccinator, n (%)	
	Yes	Yes	No	Yes	No	Yes		Yes	No	Yes	No	Yes		Yes	No	Yes	No
BCG^c^	1505	1505 (100)	N/A^d^	1477 (98.1)	28 (1.9)	372		372 (100)	N/A	369 (99.2)	3 (0.8)	1877		1877 (100)	N/A	1846 (98.3)	31 (1.7)
OPV-0^e^	1212	1212 (100)	N/A	1210 (99.8)	2 (0.2)	N/A		N/A	N/A	N/A	N/A	1212		1212 (100)	N/A	1210 (99.8)	2 (0.2)
Penta-1^f^	924	923 (99.8)	1 (0.2)	898 (97.2)	26 (2.8)	425		425 (100)	N/A	419 (98.6)	6 (1.4)	1349		1348 (99.9)	1 (0.1)	1317 (97.6)	32 (2.4)
OPV-1	924	923 (99.8)	1 (0.2)	896 (97)	28 (3)	424		424 (100)	N/A	420 (99.1)	4 (0.9)	1348		1347 (99.9)	1 (0.1)	1316 (97.6)	32 (2.4)
PCV-1^g^	924	923 (99.8)	1 (0.2)	898 (97.2)	26 (2.8)	423		423 (100)	N/A	419 (99.1)	4 (0.9)	1347		1346 (99.9)	1 (0.1)	1317 (97.8)	30 (2.2)
Rota-1^h^	924	923 (99.8)	1 (0.2)	898 (97.2)	26 (2.8)	N/A		N/A	N/A	N/A	N/A	924		923 (99.9)	1 (0.1)	898 (97.2)	26 (2.8)
IPV-1^i^	N/A	N/A	N/A	N/A	N/A	425		425 (100)	N/A	420 (98.8)	5 (1.2)	425		425 (100)	N/A	420 (98.8)	5 (1.2)
Penta-2	786	785 (99.8)	1 (0.2)	786 (100)	N/A	347		347 (100)	N/A	347 (100)	N/A	1133		1132 (99.9)	1 (0.1)	1133 (100)	N/A
OPV-2	785	784 (99.8)	1 (0.2)	785 (100)	N/A	347		347 (100)	N/A	347 (100)	N/A	1132		1131 (99.9)	1 (0.1)	1132 (100)	N/A
PCV-2	785	784 (99.8)	1 (0.2)	785 (100)	N/A	346		346 (100)	N/A	346 (100)	N/A	1131		1130 (99.9)	1 (0.1)	1131 (100)	N/A
Rota-2	784	783 (99.8)	1 (0.2)	784 (100)	N/A	N/A		N/A	N/A	N/A	N/A	784		783 (99.9)	1 (0.1)	784 (100)	N/A
Penta-3	635	634 (99.8)	1 (0.2)	635 (100)	N/A	354		354 (100)	N/A	352 (99.4)	2 (0.6)	989		988 (99.9)	1 (0.1)	987 (99.8)	2 (0.2)
OPV-3	637	636 (99.8)	1 (0.2)	635 (99.7)	2 (0.3)	354		354 (100)	N/A	353 (99.7)	1 (0.3)	991		990 (99.9)	1 (0.1)	988 (99.7)	3 (0.3)
PCV-3	637	636 (99.8)	1 (0.2)	635 (99.7)	2 (0.3)	354		354 (100)	N/A	353 (99.7)	1 (0.3)	991		990 (99.9)	1 (0.1)	988 (99.7)	3 (0.3)
IPV-2	985	982 (99.7)	3 (0.3)	653 (66.3)	332 (33.7)	406		406 (100)	N/A	355 (87.4)	51 (12.6)	1391		1388 (99.8)	3 (0.2)	1008 (72.5)	383 (27.5)
M-1^j,k^	383	380 (99.2)	3 (0.8)	362 (94.5)	21 (5.5)	144		144 (100)	N/A	137 (95.1)	7 (4.9)	527		524 (99.4)	3 (0.6)	499 (94.7)	28 (5.3)
M-2^k^	209	202 (96.7)	7 (3.3)	208 (99.5)	1 (0.5)	201		196 (97.5)	5 (2.4)	196 (97.5)	5 (2.5)	410		398 (97.1)	12 (2.9)	404 (98.5)	6 (1.5)
Total	13039	13015 (99.8)	24 (0.2)	12545 (96.2)	494 (3.8)	4922		4917 (99.9)	5 (0.1)	4833 (98.2)	89 (1.8)	17961		17932 (99.8)	29 (0.2)	17378 (96.8)	583 (3.2)

^a^WHO-recommended EPI schedule constructed by a vaccine expert, using the following criteria: BCG at ≤1-year age; OPV-0 at ≤28 days; Penta-1, OPV-1, PCV-1, Rota-1, and IPV-1 at ≥6 weeks; Penta-2, OPV-2, PCV-2, and Rota-2 at ≥10 weeks and >28 days after vaccination with Penta-1, PCV-1, OPV-1, and Rota-1; Penta-3, OPV-3, and PCV-3 at ≥4 weeks age and >28 days after vaccination with Penta-2, PCV-2, and OPV-2; IPV-2 at ≥14 weeks age (for Bangladesh, >28 days after IPV-1); measles-1 at 9 months; measles-2 at 15 months and >28 days after measles-1 vaccine (source: WHO-2020, Expanded Program on Immunization, Pakistan; WHO-2019, Fact sheet Bangladesh).

^b^n is the number of doses due for each antigen for the 6241 immunization visits recorded for 4613 children from both Pakistan and Bangladesh sites.

^c^BCG: bacille Calmette-Guérin.

^d^N/A: not applicable.

^e^OPV: oral polio vaccine.

^f^Penta: pentavalent vaccine.

^g^PCV: pneumococcal vaccine.

^h^Rota: rotavirus vaccine.

^i^IPV: inactivated polio vaccine.

^j^M: measles vaccine.

^k^In Bangladesh, measles-rubella combined vaccine is administered.

### Qualitative Analysis

#### Participant Characteristics

A total of 16 vaccinators participated in the key informant interviews (Table 3). In Pakistan, 1 (9%) vaccinator out of 11 was female, whereas in Bangladesh, the proportion of female vaccinators was 80% (4/5). On average, the participants had been working as vaccinators for 7.6 (SD 8.4) years, and the mean age was 32 (SD 9.3) years. In Pakistan, nearly half (6/11, 54%) of all the vaccinators interviewed had >12 years of education, whereas in Bangladesh, this proportion was 100% (5/5).

We extracted 3 major themes through thematic analysis of in-depth interviews conducted with the vaccinators.

**Table 3:** Characteristics of vaccinators interviewed in Pakistan and Bangladesh.

Characteristics			Participants				
			Pakistan (n=11)		Bangladesh (n=5)		Total (N=16)
Female, n (%)			1 (9.1)		4 (80)		5 (31.3)
**Years of education, n (%)**							
	≤12	5 (45.5)		N/A^a^		5 (31.3)	
	>12	6 (54.5)		5 (100)		11 (68.8)	
Age (years), mean (SD)			32.0 (9.0)		32.2 (11.1)		32.0 (9.3)
Years of experience, mean (SD)			8.1 (9.8)		6.6 (4.7)		7.6 (8.4)

^a^N/A: not applicable.

#### Theme 1: Understanding of iDSS

All (16/16, 100%) vaccinators appreciated the iDSS module for calculating the age-appropriate vaccine schedules for each visit. They relayed good knowledge of iDSS functionality and 69% (11/16) believed that they could effortlessly explain the app features to their fellow colleagues. Furthermore, most (13/16, 81%) vaccinators were aware of the color scheme and were able to interpret it correctly:

The color codes give us indications; vaccines that are supposed to be administered on current visit have different color codes, and already vaccinated have a different color code.

Vaccinator 3

On assessing the knowledge about key variables on which the iDSS algorithm functions, very few vaccinators (6/16, 37%) were aware that the iDSS predicted future dates using the variables of date of birth, age, and immunization history*.*

#### Theme 2: Functionality of iDSS

##### Automatic Construction of Age-Appropriate Vaccine Schedules

All the vaccinators (16/16, 100%) appreciated the instantaneous calculation of age-appropriate schedules using the iDSS. They noted and were able to explain the significance of dates that appeared against each vaccine that was due on the current visit, given previously, or needed to be scheduled for an upcoming visit:

When we enroll a child, it automatically tells which vaccine has to be administered and the dates are same as the dates we used to calculate manually.

Vaccinator 10

iDSS is an easy app to use. Precisely I can say, I don’t need to think about dates or about weekend. It automatically generates dates, and we can update our record book and EPI card easily without error.

Vaccinator 13

##### Accuracy in Generating Age-Appropriate Vaccine Schedules

The vaccinators conveyed mixed responses regarding the perceived accuracy of the iDSS algorithm across both the sites. Less than half (7/16, 44%) of the vaccinators stated that they found no discrepancies in the schedules constructed by the iDSS, whereas others stated discrepancies that contradicted their routine practices.

Despite endorsing the accuracy, 62% (10/16) participants across both sites were confused about the age-appropriate administration of IPV and measles vaccines. For instance, 54% (6/11) vaccinators in Pakistan were unsure about administering the IPV vaccine due at the age of 14 weeks according to the national immunization schedule. Some vaccinators believed that IPV should be administered with the third dose of Penta, irrespective of the age of the child, and hence considered iDSS to have scheduled the IPV dose inaccurately:

It happens that if a child’s age is let’s say 3 months or 3.5 months and he comes in for BCG, application shows IPV to be given as well at 3.5 months, but we don’t do so and schedule IPV for future.

Vaccinator 1

One of the participants from Pakistan (1/11, 9%) also questioned the interval between the 2 doses of measles, particularly for children who were behind their routine schedules:

We have some confusions, for example if a child’s age is 12 months, then it (iDSS) gives date randomly and does not keep a gap of 3 months between two doses.

Vaccinator 8

Vaccinators in Bangladesh (3/5, 60%) were unsure about the scheduling of upcoming vaccines that are not dependent on previous doses (for instance, the first dose of measles vaccine is not dependent on pentavalent-3 dose and should be administered when a child turns 9 months). Vaccinators believed that the measles vaccine should be “locked” on the iDSS (ie, not allowed to be given) until the child receives the pentavalent-3 dose. The same logic was applied for the IPV-2 dose due at the age of 14 weeks in Bangladesh:

Why do I have to schedule Measles and Rubella during Penta-2 vaccination? Isn’t it supposed to be given only after Penta-3–IPV 2 schedule?

Vaccinator 14

#### Theme 3: Usability of iDSS

##### Utility of the iDSS for Vaccinators

More than half (9/16, 56%) of the vaccinators stated that the iDSS is easy to use and 69% (11/16) emphasized its need as it accurately constructs age-appropriate vaccine schedules quickly, saving time. Vaccinators highlighted that the iDSS facilitates decisions regarding whether the child should receive vaccination or not, eventually reducing MOVs:

Best thing are the dates/schedules that are automatically generated. It is less time consuming and the vaccines that are needed to be administered are ticked.

Vaccinator 1

Initially, we were tallying dates that we predicted with the dates shown in iDSS. We found some mismatch in the dates, but it was our mistake mostly, because we did not do [the calculations] properly. Some days were missed by us.

Vaccinator 9

Almost one-fifth (3/16, 19%) of the vaccinators thought that iDSS would be more useful for outreach activities in which they often encounter children who have missed their routine vaccination doses:

Though our country has a wide range of EPI centers, during our outreach activities we try to reach defaulter children and ensure to give them all required vaccines displayed as due vaccines on this app [iDSS].

Vaccinator 13

Vaccinators (6/16, 37%) also suggested that iDSS should serve as a channel to digitalize immunization information systems and replace the conventionally used manual calculation methods for constructing vaccine schedules:

Our country is going to digitalize the vaccination system and we are still dependent on the old hardcopy methods. It was needed in the past since we have started EPI activity. It will assist in data centralization.

Vaccinator 13

##### Overall Feedback on iDSS

Overall, the vaccinators were satisfied with the app functionality and provided positive feedback about the app:

In my experience iDSS is far better than our current system. Moreover, auto generation of vaccine schedules helps us to enter data within no time.

Vaccinator 14

Vaccinators (7/16, 44%) suggested that the color scheme should be reconsidered:

There are no issues with color scheme and its well understood; however, if red could be replaced by any other color it makes us feel better. You know red is used to indicate danger.

Vaccinator 4

All vaccinators admired the current iDSS interface in terms of color scheme, text font and size, and design. Vaccinators mentioned that there was room for improvement, but they did not provide specific details of the features that needed to be changed:

No this is okay, we don’t have any issues in that, the design is okay and so is the text.

Vaccinator 8

#### Theme 4: Challenges With the Manual Calculation of Catch-up Schedules

As part of their routine work, vaccinators were mainly dependent on vaccination cards or parental recall to infer the date of birth of children and their vaccination history to manually calculate immunization schedules:

If someone has lost the EPI card then we try to figure out through a recall method and ask the parent to tell us when the child gets the first vaccine. Then we ask for the date of birth, which they usually don’t know, and tell us the age of the child. In this way, based on verbal discussion and age of child, for example, they say 2 or 3.5 months, we take the risk and vaccinate the child for 2nd or 3rd dose accordingly.

Vaccinator 11

For follow-up visits, vaccinators mentioned that the usual practice was to provide a calendar date with a 1-month gap for the next dose instead of following the standard 28-day interval between scheduling future doses as per the WHO-recommended EPI schedule guidelines. Vaccinators mentioned that to schedule doses with a 28-day interval, they had to refer to calendar and count the days to find the exact dates. A few times, they did not account for weekends when scheduling the subsequent visits. Vaccinators found iDSS useful in providing schedules automatically for subsequent visits that were in line with the WHO EPI guidelines:

Previously, we had to see the dates one by one and calculate it, now the benefit is we just enter data and get dates automatically. It also shows the schedule for my next visit.

Vaccinator 5

## Discussion

### Principal Findings

Our study has demonstrated the diagnostic accuracy and feasibility of iDSS to accurately schedule age-appropriate vaccination doses across a multicountry LMIC setting. We also demonstrated the potential of iDSS to reduce MOVs caused by poor vaccinator adherence to standard immunization schedules and the interpretation of complex immunization schedules. Our study reports positive feedback received from a diverse set of vaccinators with varying levels of education and experience on the usability and acceptability of the iDSS.

Overall, the iDSS had higher accuracy in constructing age-appropriate immunization schedules as per the WHO-recommended EPI guidelines compared with vaccinators. Our study highlights the issue of missed vaccine doses despite children making contact with vaccinators. Reducing MOVs caused by complexity and changes in EPI schedules can improve immunization coverage, timeliness, and equity [14]. One study from the United States estimated that the potentially achievable vaccination coverage for 4+Diphtheria, tetanus, and acellular pertussis vaccine, 4+PCV, and full series of *Haemophilus influenzae* type b for children aged 19 to 35 months would have been 90% if missed opportunities in the administration of these vaccines were eliminated [26]. From an equity perspective, iDSS can enable children from disadvantaged backgrounds, who are already delayed on their vaccination, to catch-up with the rest through accurate administration of all due vaccine doses when they encounter a vaccinator.

Our findings show that the highest MOVs across Pakistan and Bangladesh sites were for polio and measles vaccines, as 27.5% (383/1391) and 5.3% (28/527) of all due immunization doses, respectively, were missed by vaccinators. A systematic review of MOVs across LMICs also reported the prevalence rate of MOVs with regard to polio vaccines to range from 13.4% to 46.7% [2]. According to the WHO-recommended EPI schedule, Pakistan and Bangladesh administer the IPV vaccine to all children at 14 weeks. However, feedback from vaccinators across both sites revealed that vaccinators considered it to be given alongside vaccines administered with Penta-3 (irrespective of age). This practice delayed the IPV vaccination, as the Penta, OPV, and PCV vaccines depend on previous doses and are often administered beyond 14 weeks of age [27]. These MOVs, especially for polio vaccines, are alarming, as they impede global efforts to eradicate polio. Pakistan remains one of the last 2 polio-endemic countries with a substantial surge in the number of polio cases since 2017 (147 cases in 2019 vs 8 in 2017) [28], putting the country on a failing trajectory and the broader Global Polio Eradication Initiative at risk. Similarly, the measles incidence in the country increased from 24.6 cases per million to 80.4 per million between 2000 and 2018 [29].

Although previous studies have reported examples of EIR in LMIC settings [30,31], not all of them have an in-built immunization scheduling or decision support system [32-34]. Our study adds to this important area by providing evidence of the diagnostic accuracy of a stand-alone mobile-based iDSS implemented across a multicountry LMIC setting. Our results are in line with findings reported from high-income countries where iDSS has improved the scheduling of vaccinations. A hospital-based study in the United States that implemented a computer-based clinical decision support algorithm demonstrated an increase in the tetanus, diphtheria, pertussis vaccination in postpartum women [35]. Our results corroborate this finding, making a strong case for high diagnostic accuracy of iDSS technology in LMIC settings. In addition, the end-user feedback from a diverse set of vaccinators, who varied in terms of gender, education, age, and experience, also confirms the utility and functionality of iDSS, its acceptance, and user satisfaction; however, interviews with vaccinators in our study did reveal that only about half (9/16, 56%) of them found the iDSS easy to use, presumably because of maintaining paper-based and electronic records simultaneously in the study. Switching to electronic records completely is likely to address this concern [14].

In contrast to most web-based iDSS developed and used in high-income countries, the iDSS in this study is packaged in the form of an API that is interoperable with health information systems and allows flexibility of deployment. As EIRs continue to be adapted, incorporating iDSS through APIs should be considered, in line with established standards such as the Pan American Health Organization EIR guidelines [36]. API-based iDSS such as the one used in this study also allows flexibility to adjust to multicountry EPI schedules that change frequently and can also adapt to cosmetic user interface changes such as varying languages and displays.

Integration of an iDSS module in LMIC Immunization Programs can, therefore, yield enormous benefits, not only in terms of reducing MOVs but also in designing accurate catch-up regimens to ensure universal immunization. This is particularly relevant in the context of the huge numbers of children who have missed their vaccine doses during the COVID-19 pandemic and associated lockdowns [18]. Equipping vaccinators with iDSS technology can help ensure that these children are effectively immunized for all due vaccines at subsequent visits, thereby maintaining optimal immunity levels and reducing the likelihood of secondary vaccine-preventable disease outbreaks.

### Strengths and Limitations

A major strength of our study was the demonstration of the feasibility of the iDSS across 2 different LMIC settings, with varying EPI schedules, infrastructure, coverage levels, vaccinator education, experience, demographics, and immunization-related challenges. One limitation of our study was that the evidence of MOVs generated in this study only accounted for MOVs because of inaccurate vaccination schedules constructed by vaccinators and hence would be an underestimation of the overall MOV prevalence in our study sites.

### Conclusions

The iDSS has high diagnostic accuracy for scheduling age-appropriate vaccinations and reducing MOVs; and high acceptability among vaccinators regardless of gender, education, and experience. The iDSS boasts a variety of features for easy adaptability and replicability across LMIC settings. The evidence generated from this study demonstrates the prevalence of MOVs, especially for measles and polio vaccines. iDSS can increase immunization coverage, timeliness, and equity by eliminating these MOVs and help design accurate catch-up regimens to ensure universal immunization, especially in the aftermath of the COVID-19 pandemic. The findings from this study provide the impetus for rigorously evaluating the impact of iDSS through a randomized controlled trial and paving the way for a scaled implementation of this tool across LMICs.

## Appendix

Multimedia Appendix 1 Immunization decision support system supplementary information.

## Abbreviations

API: application programming interface
BCG: bacille Calmette-Guérin
EIR: electronic immunization registry
EPI: Expanded Programme on Immunization
iDSS: immunization decision support system
IPV: inactivated polio vaccine
LMIC: low- and middle-income country
MOV: missed opportunity for vaccination
MR: measles-rubella
OPV: oral polio vaccine
PCV: pneumococcal vaccine
Penta: pentavalent vaccine
Rota: rotavirus vaccine
WHO: World Health Organization

## Data Availability

Deidentified participant data and data dictionary are available to any researcher upon reasonable request.

## References

[1] (2022). Diphtheria tetanus toxoid and pertussis (DTP) vaccination coverage. United Nations Children’s Fund, World Health Organization.

[2] Sridhar S, Maleq N, Guillermet E, Colombini A, Gessner BD (2014). A systematic literature review of missed opportunities for immunization in low- and middle-income countries. Vaccine.

[3] Reducing Missed Opportunities for Vaccination (MOV). World Health Organization.

[4] Adamu AA, Sarki AM, Uthman OA, Wiyeh AB, Gadanya MA, Wiysonge CS (2019). Prevalence and dynamics of missed opportunities for vaccination among children in Africa: applying systems thinking in a systematic review and meta-analysis of observational studies. Expert Rev Vaccines.

[5] Restrepo-Méndez MC, Barros AJ, Wong KL, Johnson HL, Pariyo G, Wehrmeister FC, Victora CG (2016). Missed opportunities in full immunization coverage: findings from low- and lower-middle-income countries. Glob Health Action.

[6] Li AJ, Tabu C, Shendale S, Okoth PO, Sergon K, Maree E, Mugoya IK, Machekanyanga Z, Onuekwusi IU, Ogbuanu IU (2020). Qualitative insights into reasons for missed opportunities for vaccination in Kenyan health facilities. PLoS One.

[7] Sambala EZ, Uthman OA, Adamu AA, Ndwandwe D, Wiyeh AB, Olukade T, Bishwajit G, Yaya S, Okwo-Bele JM, Wiysonge CS (2018). Mind the Gap: what explains the education-related inequality in missed opportunities for vaccination in sub-Saharan Africa? Compositional and structural characteristics. Hum Vaccin Immunother.

[8] Manandhar P, Wannemuehler K, Danovaro-Holliday MC, Nic Lochlainn L, Shendale S, Sodha SV (2023). Use of catch-up vaccinations in the second year of life (2YL) platform to close immunity gaps: a secondary DHS analysis in Pakistan, Philippines, and South Africa. Vaccine.

[9] Kahane SM, Watt JP, Newell K, Kellam S, Wight S, Smith NJ, Reingold A, Adler R (2000). Immunization levels and risk factors for low immunization coverage among private practices. Pediatrics.

[10] Cohen NJ, Lauderdale DS, Shete PB, Seal JB, Daum RS (2003). Physician knowledge of catch-up regimens and contraindications for childhood immunizations. Pediatrics.

[11] Ogbuanu IU, Li AJ, Anya BP, Tamadji M, Chirwa G, Chiwaya KW, Djalal ME, Cheikh D, Machekanyanga Z, Okeibunor J, Sanderson C, Mihigo R (2019). Can vaccination coverage be improved by reducing missed opportunities for vaccination? Findings from assessments in Chad and Malawi using the new WHO methodology. PLoS One.

[12] Smalley HK, Keskinocak P, Engineer FG, Pickering LK (2011). Universal tool for vaccine scheduling: applications for children and adults. Interfaces.

[13] Engineer FG, Keskinocak P, Pickering LK (2009). OR practice—catch-up scheduling for childhood vaccination. Oper Res.

[14] Zaidi S, Shaikh SA, Sayani S, Kazi AM, Khoja A, Hussain SS, Najmi R (2020). Operability, acceptability, and usefulness of a mobile app to track routine immunization performance in rural Pakistan: interview study among vaccinators and key informants. JMIR Mhealth Uhealth.

[15] Li L, Ackermann K, Baker J, Westbrook J (2020). Use and evaluation of computerized clinical decision support systems for early detection of sepsis in hospitals: protocol for a scoping review. JMIR Res Protoc.

[16] Kihlgren A, Svensson F, Lövbrand C, Gifford M, Adolfsson A (2016). A Decision support system (DSS) for municipal nurses encountering health deterioration among older people. BMC Nurs.

[17] Sim I, Gorman P, Greenes RA, Haynes RB, Kaplan B, Lehmann H, Tang PC (2001). Clinical decision support systems for the practice of evidence-based medicine. J Am Med Inform Assoc.

[18] Ye J (2020). The role of health technology and informatics in a global public health emergency: practices and implications from the COVID-19 pandemic. JMIR Med Inform.

[19] Murthy N, Rodgers L, Pabst L, Fiebelkorn AP, Ng T (2017). Progress in childhood vaccination data in immunization information systems - United States, 2013-2016. MMWR Morb Mortal Wkly Rep.

[20] Zhu VJ, Grannis SJ, Rosenman MB, Downs SM (2009). Implementing broad scale childhood immunization decision support as a web service. AMIA Annu Symp Proc.

[21] (2019). Pakistan demographic and health survey 2017-18. NIPS, ICF.

[22] (2015). Success factors for women’s and children’s health: Bangladesh. World Health Organization.

[23] (2019). Pakistan Social and Living Standard Measurement Survey (PSLM). Statistics Division, Government of Pakistan.

[24] Sajid E (2020). Teledensity nearing 100% in Bangladesh. The Business Standard.

[25] Sarker AR, Akram R, Ali N, Sultana M (2019). Coverage and factors associated with full immunisation among children aged 12-59 months in Bangladesh: insights from the nationwide cross-sectional demographic and health survey. BMJ Open.

[26] Zhao Z, Smith PJ, Hill HA (2016). Evaluation of potentially achievable vaccination coverage with simultaneous administration of vaccines among children in the United States. Vaccine.

[27] Akmatov MK, Mikolajczyk RT (2012). Timeliness of childhood vaccinations in 31 low and middle-income countries. J Epidemiol Community Health.

[28] (2021). Polio Cases in Provinces. Pakistan Polio Eradication Programme.

[29] Mere MO, Goodson JL, Chandio AK, Rana MS, Hasan Q, Teleb N, Alexander Jr JP (2019). Progress toward measles elimination - Pakistan, 2000-2018. MMWR Morb Mortal Wkly Rep.

[30] Secor AM, Mtenga H, Richard J, Bulula N, Ferriss E, Rathod M, Ryman TK, Werner L, Carnahan E (2022). Added value of electronic immunization registries in low- and middle-income countries: observational case study in Tanzania. JMIR Public Health Surveill.

[31] Seymour D, Werner L, Mwansa FD, Bulula N, Mwanyika H, Dube M, Taliesin B, Settle D (2019). Electronic immunization registries in Tanzania and Zambia: shaping a minimum viable product for scaled solutions. Front Public Health.

[32] Pancholi J, Birdie R, Guerette J, Chritz S, Sampath V, Crawford J (2020). Landscape analysis of electronic immunization registries; lessons learned from a landscape analysis of EIR implementations in low and middle income countries. VillageReach.

[33] Kombian H (2017). Electronic immunization registry and tracking system in Sierra Leone. eHealth Africa.

[34] Oliver-Williams C, Brown E, Devereux S, Fairhead C, Holeman I (2017). Using mobile phones to improve vaccination uptake in 21 low- and middle-income countries: systematic review. JMIR Mhealth Uhealth.

[35] Trick WE, Linn ES, Jones Z, Caquelin C, Kee R, Morita JY (2010). Using computer decision support to increase maternal postpartum tetanus, diphtheria, and acellular pertussis vaccination. Obstet Gynecol.

[36] (2017). Electronic immunization registry: practical considerations for planning, development, implementation and evaluation. Pan American Health Organization.

