# Supplementary material for: Evaluation of a Mobile-Based Immunization Decision Support System for Scheduling Age-Appropriate Vaccine Schedules for Children Younger Than 2 Years in Pakistan and Bangladesh: Lessons From a Multisite, Mixed Methods Study
Source: JMIR Pediatr Parent. 2023 Feb 17;6:e40269. doi: 10.2196/40269 (PMC9984999; doi:10.2196/40269)
Supplement: Multimedia Appendix 1 [file pediatrics_v6i1e40269_app1.docx]

**Supplementary Material**

**Overview of Immunization Decision Support System (iDSS) for scheduling age-appropriate vaccines**

**Introduction:**

The iDSS is an android-based application designed to formulate age-appropriate immunization schedules (including the vaccines due at the current visit and those to be scheduled) tailored to the child and the respective country’s EPI schedule. The application was developed and packaged as an application programming interface (API), making it interoperable with other applications, such as other mobile-based and web-based applications as well as electronic immunization registries.

**Features:**

The iDSS has the following features:

1. An algorithm-based construction of an age-appropriate immunization schedule that takes into account the child’s age or date of birth, immunization history, inter-dose vaccine gaps, center specific immunization days, and holidays
2. A display feature for age-appropriate vaccination schedule using a preset color-coded scheme
3. Scanning of QR codes for maintaining unique records of individual children
4. Capturing and retaining demographic information of the child that could be fetched on a follow-up visit
5. Search option based on multiple parameters to draw child’s information in case of missed QR code or vaccination card
6. Web-based dashboard for real-time metadata management and reporting
7. Login for individual users in the mobile-based application and the web dashboard to ensure data security
8. An offline mode
9. Ability to add multi-country immunization schedules
10. Capacity to function using multiple languages


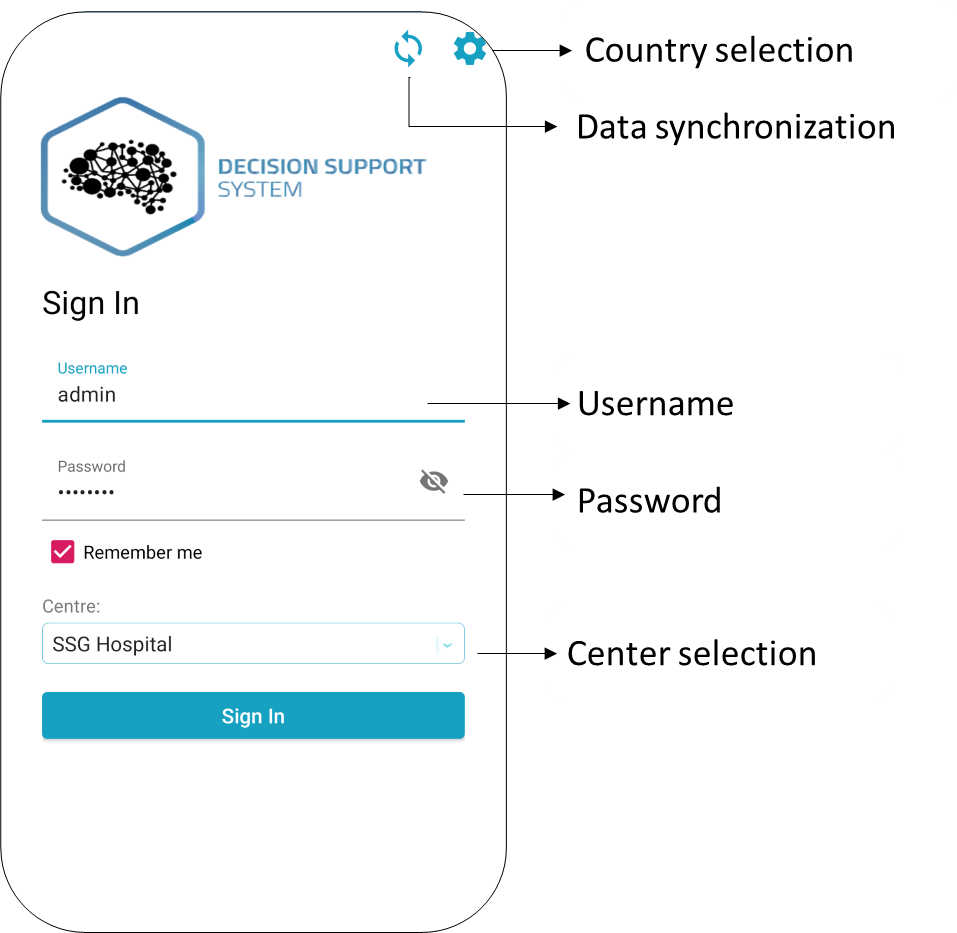


Figure 1: Log in screen

# **Components:**

The iDSS application includes an input and output component

In the input component, the user enters the information into the application (including the child’s date of birth/age and immunization history). The iDSS uses this information and the underlying algorithm to generate an age-appropriate vaccination schedule, including antigens that need to be administered on the current visit. To construct catchup schedules, the iDSS considers the child’s date of birth or age and immunization history. Moreover, it observes variances between the recorded vaccination details and default schedule, keeping a lax time of ±10 days.

In the output component, iDSS display features shows the immunization schedule constructed by the application. The display screen has a built-in layout that shows the list of vaccines and a color-coded scheme to guide the user regarding the timing of the vaccine administration. Each color indicates a particular time which is as follows:

1. Red: vaccines that must be administered on the current visit. These can be scheduled on another day if they cannot be administered on the current visit for any reason
2. Yellow: optional vaccines can be administered on the current visit
3. Blue: vaccines available for scheduling on the subsequent vaccination visit
4. Locked vaccines represent antigens that cannot be administered as the pre-requisite vaccine has not been administered yet

# **Functionality:**

The basic functionality of the iDSS application for the purpose of the current study includes logging in, country selection, site (immunization centre) selection, assessing the child’s eligibility (for the study), enrollment or follow-up of the child, and accessing child’s vaccination records. The process begins by the user using pre-generated login credentials to log in to the mobile application and selecting his/her country to ensure that the iDSS algorithm follows the immunization schedule of that particular country. The child is then screened as per the eligibility criteria of the study. If the child is eligible for the study, he/she is enrolled into the iDSS using the child enrolment form. In case of a follow-up visit of a child, the iDSS proceeds to the follow-up form.

On the first visit, after being assessed for eligibility, a 10-digit unique QR code is assigned for uniquely identifying and tracking the record of the child. The user then captures detailed biographic and demographic information of the child. This information is fetched on the subsequent follow-up visits by scanning of unique QR code provided on the first visit.

The algorithm to determine the age-appropriate vaccination schedule accounts for the child’s age or date of birth, prerequisite vaccine check, inter-dose gap, as well as public holidays and non-operational days for specific antigens in the particular immunization center. Upon inputting the required information (child’s date of birth/age and immunization history), the iDSS displays the vaccine schedule with a color-coded scheme on the display screen. After the user has administered the vaccines on the current visit, he/she updates the vaccination record by indicating the correct status against the vaccines proposed by the iDSS (vaccinated, scheduled, not available, not recommended) and date of vaccination for each antigen. On the follow-up visit, the vaccination schedule automatically shows vaccines that should be administered on the current visit.

The web-based dashboard for administrative data management and reporting is built using Django, a python-based web framework. The data is stored using PostgreSQL. The dashboard is accessible only to the designated team members with pre-generated user credentials. The data communication between the server and the mobile application is done using encrypted channels over HTTPS with key pass authentication. The application is fully functional offline, allowing users to work without an internet connection and enable data synchronization were in when connectivity is available.

**
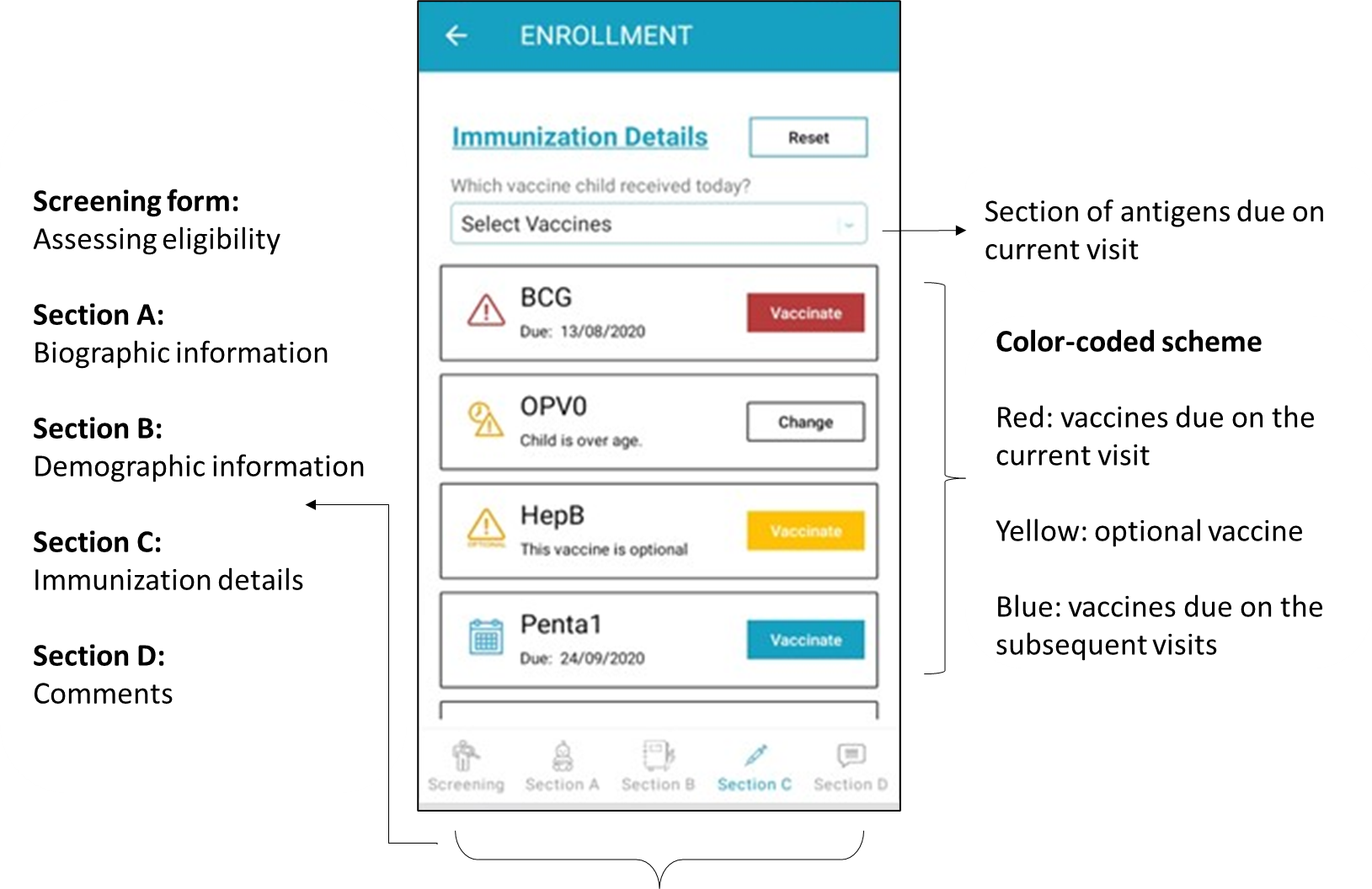
**

Figure 2: Features of iDSS application with color-coded scheme
